# Exclusion of eleven candidate genes for ocular melanosis in cairn terriers

**DOI:** 10.1186/1477-5751-12-6

**Published:** 2013-03-01

**Authors:** Paige A Winkler, Joshua T Bartoe, Celeste R Quinones, Patrick J Venta, Simon M Petersen-Jones

**Affiliations:** 1 Department of Small Animal Clinical Sciences, College of Veterinary Medicine, Michigan State University, 48824, East Lansing, MI, USA; 2 Department of Microbiology and Molecular Genetics, Michigan State University, 48824, East Lansing, MI, USA

**Keywords:** Dog, Glaucoma, Ocular melanosis, Pigmentary glaucoma, Candidate gene

## Abstract

**Background:**

Ocular melanosis of Cairn terrier dogs is an inherited defect characterized by progressive pigmentation of both eyes which can result in glaucoma and blindness. Pedigree analysis suggests the trait has an autosomal dominant mode of inheritance. We selected 11 potential candidate genes and used an exclusion analysis approach to investigate the likelihood that one of the candidate gene loci contained the Cairn terrier-ocular melanosis locus.

**Results:**

Two polymorphic loci were identified within or close to each candidate gene. Genotyping of at least 10 ocular melanosis Cairn terriers for each marker showed that there was no single shared allele for either of the two polymorphic markers identified in *ASIP*, *COMT*, *GPNMB*, *GSK3B*, *LYST*, *MC1R*, *MITF*, *SILV*, *TYR*, *TYRP1,*and *TYRP2*. This is strong evidence to exclude each locus as the site of the ocular melanosis mutation (probability of a false exclusion calculated for each gene ranged from 1.59 × 10^-4^ to 1 × 10^-9^).

**Conclusions:**

None of the 11 potential candidate genes selected are likely to be the gene locus for ocular melanosis in Cairn terriers.

## Background

Ocular melanosis (OM) is a hereditary condition in the Cairn terrier breed of dog that is characterized by proliferation and migration of pigmented cells starting in the anterior uvea of both eyes. The pigmented cells result in a diffuse expansion of the root of the iris and are also shed into the aqueous humor. They accumulate in the anterior sclera which develops pigment plaques that progressively increase in size, possibly reaching that site via the conventional aqueous drainage pathways [1,2]. Eventually the aqueous drainage pathways become blocked with pigment results in secondary glaucoma and vision loss. The pigmented cells appear to be primarily melanocytes although some admixture with melanophages (macrophages that have ingested melanosomes) does occur [3]. The pigmented cells also reach the posterior segment of the eye and over time can be seen to slowly migrate into and obscure the tapetum [2]. The posterior sclera also becomes pigmented and pigment can be found in the meninges around the optic nerve. Transformation to develop solid melanocytic tumors occurs in some cases and was reported in 3 dogs in a series of 114 ocular melanosis affected Cairn terriers [3].

Cairn terrier OM shares some similarities with human pigment dispersion syndrome (PDS). PDS patients accumulate pigment within the trabecular meshwork, and a subset (approximately 35% by 35 years following PDS diagnosis) develop pigmentary glaucoma (PG) [4]. This condition accounts for 1–1.5% of human glaucoma cases seen in the Western world [5]. Although the PDS causative mutation/s has yet to be identified, the phenotype has been linked to the telomere region (7q35-q36) of human chromosome 7 [6].

Rodent models with pigment changes similar to human PDS and PG, and pseudoexfoliation have been described. In the DBA/2 J mouse, mutations in *GPNMB* and *TYRP1* result in iris pigment dispersion and iris stromal atrophy respectively and mice homozygous for recessive alleles at both loci have earlier onset and more severe disease [7,8]. Clinically this mouse model closely resembles Cairn terrier OM. More recently a mutation in *LYST* has been shown to cause iris transillumination defects in mice [9].

OM in Cairn terriers represents a potentially important large animal model for investigation of human PDS and PG. Pedigree analysis indicates that Cairn terrier OM is likely to be inherited as an autosomal dominant trait [2]. We tested the hypothesis that one of 11 candidate genes has suffered a mutation that causes OM phenotype by examination of allele sharing of genetic markers close to or within each gene. Using an association-based form of exclusion analysis (as opposed to pedigree-based exclusion analysis), we provide evidence that strongly supports the exclusion of these 11 candidate genes from association with OM in the Cairn terrier.

## Results

### Candidate genes

Eleven potential candidate genes for ocular melanosis were selected: agouti signaling protein (*ASIP*), catechol-o-methyltransferase (*COMT*), glycoprotein NMB (*GPNMB*), glycogen synthase kinase 3-beta (*GSK3B*), lysosomal trafficking regulator (*LYST*), melanocortin 1 receptor (*MCIR*), microphthalmia transcription factor (*MITF*), silver (*SILV*), tyrosinase (*TYR*), tyrosinase related protein 1 (*TYRP1*), and Tyrosinase related protein 2 (*TYRP2*). For each gene two polymorphic markers were identified (11 single nucleotide polymorphisms (SNPs), 9 microsatellites (MS), 2 insertion/deletions (in/dels)) that could be used to test for association of the candidate locus with Cairn terrier OM. Details of the candidate genes and markers are shown in Table 1.

**Table 1:** Candidate genes for Cairn terrier ocular melanosis with markers, chromosomal positions, and PCR primers

**Gene, Protein, Position**^**a**^	**Marker**^**b **^**[Restriction enzyme]**	**Marker Position**^**c**^	**Distance (kb)**^**d**^	**P values**^**e**^
*LYST* Lysosomal Trafficking Regulator chr4:7,128,220-7,294,031	Microsat 1 (TTTTC)18(TTTTC)11	1^§ ^	345	1.00 × 10^-5^
	Microsat 2 (GAAA)5 (GAAA)13	2^§ ^	368	
*MC1R* Melanocortin 1 receptor chr5:66,692,398-66,693,344	SNP 1 (BICF2P987741)	2^∆ ^	560	1.34 × 10^-5^
	SNP 2 (BICF2S23213233) [SsiI]	1^∆ ^	24	
SILV Silverchr10:3,273,996-3,279,352	Microsat 1 (GAAA)7(GAAA)17	2^§ ^	60	1.00 × 10^-5^
	Microsat 2 (TTTC)15(TTCC)13	1^§ ^	226	
*TYRP1* Tyrosinase related protein 1 chr11:36,344,712-36,361,793	SNP 1 (BICF2S23051528) [BC1I]	1^∆ ^	224	2.15 × 10^-5^
	In/Del	2^∆ ^	96	
*GPNMB* Glycoprotein NMB chr14:39,877,810-39,905,589	SNP 1 (BICF2P753624) [HpyCH4V]	M1°	26	1.59 × 10^-4^
	SNP 2 (BICF2P134952)	2^∆ ^	1200	
*MITF* Microphthalmia transcription factor chr20:24,853,657-24,884,775	SNP 1 (BICF2G630233682) [BspHI]	2^∆ ^	73	3.80 × 10^-5^
	SNP 2 (BICF2S23248988)	1^∆ ^	521	
TYR Tyrosinasechr21:13,797,070-13,891,317	Microsat 1 (GAAA)17(GGAA)20(GAAA)10	2^§ ^	428	1.00 × 10^-9^
	Microsat 2 (TTTC)10(TTTC)4(TTTC)13	1^§ ^	70	
*TYRP2* (*DCT*) Tyrosinase related protein 2 chr22:48,219,817-48,254,000	SNP 1 (BICF2S23137809) [sequenced]	M2°	33	8.47 × 10^-4^
	SNP 2 (BICF2P452919) [RsaI]	M1°	8	
*ASIP* Agouti signaling protein chr24:26,327,360-26,366,307	SNP 1 (BICF2P1186810) [MesI]	1^∆ ^	340	3.57 × 10^-5^
	In/Del	2^∆ ^	105	
*COMT* Catechol-O-Methyltransferase chr26:32,426,959-32,432,523	SNP 1 (BICF2S22923369) [ApaLI]	2^∆ ^	82	8.20 × 10^-5^
	Microsat 1 (TTTA)15	1^§ ^	676	
*GSK3B* Glycogen Synthase Kinase 3-Beta chr33:26,516,949-26,699,712	Microsat 1 (CTATT)14	M2*	146	1.00 × 10^-5^
	Microsat 2 (TTTA)13	M2*	143	

Key:

a. Genes are listed in chromosomal order as obtained from the May 2005 build of the canine reference genome (UCSC Genome Browser: http://genome.ucsc.edu/), with the encoded proteins provided under the gene names.

b. Microsatellite-based markers are shown with the repeat type and number of perfect repeats present in the canine reference genome. Repeat blocks are separated by one to several nucleotides that do not match the perfect repeat. SNPs are listed with Broad Institute CanFam 2.0 SNP designation ( http://www.broadinstitute.org/mammals/dog). One marker for ASIP is an in/del of an undefined but variable nature. The restriction enzyme used for PCR-restriction enzyme method of genotyping SNPs is shown in square brackets. Note some were genotyped by sequencing.

c. The location of each marker is given with respect to coding region of each gene as seen in the 2005 canine reference genome on UCSC Genome Browser. Designations are upstream (1) or downstream (2) of the gene start site, or within the gene upstream of the exact midpoint (M1) or to the downstream of the midpoint (M2).

d. Distances are given from each marker to furthest end of the gene from that marker.

e. Each P value is the probability (combined probability of the two markers) of falsely excluding the true causative gene (see Additional file 2: Supplementary Methods for an example of how this was calculated). The method used for each marker is indicated in the column showing marker position: § used microsatellite mutation rate, ∆ used rate of recombination between marker and coding region, o used rate of recombination from marker to nearest end of the coding region (probability of the recombination WITHIN the gene), * Clark’s method for haplotype analysis [10].

### The exclusion analysis

Exclusion analysis was performed under several assumptions that are considered to be robust for canine Mendelian disorders; (1) all affected dogs share the same causal mutation, which is identical by descent due to a founder event (very likely, based upon previous results for common Mendelian mutations in purebred dogs; see Discussion), (2) no recombination has occurred between the marker and the mutation since the time the unique mutational event occurred (markers are very close to the gene and the probability of a rare event is calculated and presented), and (3) no new mutations have occurred in either the marker or the gene (also included in the probability calculation for a false exclusion). It is important to understand that, for the purpose of exclusions, only affected dogs are needed. This is analogous to exclusion in paternity testing. If an accused male does not share marker alleles with the baby, he cannot be the father; knowing or not knowing the marker allele frequencies in the population has no impact on the exclusion. It is only necessary to have allele frequency information when the alleles in the offspring are consistent with those of the accused male in order to provide a probability statement on how likely this match is compared to other possible males in the population. We sought to exclude most candidate genes as quickly and affordably as possible because only one gene can underlie a Mendelian disorder due to a founder event and the rest of the candidate genes are “innocent” with respect to the disease under examination. An advantage to this approach is the cost savings obtained because it is not necessary to genotype unaffected controls. Controls only need to be genotyped if a gene cannot be excluded, in order to obtain positive evidence of an association between a locus and the disease phenotype. Note also that penetrance is not an issue because all Cairn terriers genotyped are affected and must share the necessary allele.

With a likely dominant mode of inheritance, markers within or close to the gene harboring the causal mutation (or a necessary locus) would have at least one shared allele between affected dogs. For each of the 22 markers genotyped there was no shared single allele between the OM-affected dogs thus providing evidence that none of the candidate genes underlie OM (Additional file 1: Table S1 and Table S2). We then calculated the probability of falsely excluding the candidate genes: the p values ranged from 1.59 × 10^-4^ to 1.0 × 10^-9^ (Table 1 and Additional file 2), making it very unlikely that any one the 11 candidate genes tested contains the OM mutation.

## Discussion

Linkage-based and sequence-based exclusion analyses have been used in many studies to eliminate candidate genes hypothesized to underlie inherited phenotypes in dogs (for example: [11-13]). With late-onset conditions such as ocular melanosis compiling pedigrees (DNA and phenotypes from multiple offspring and at least one parent) can be difficult because it may not be possible to locate the siblings or the parents of affected animals, or they may already be dead. Sequencing of candidate genes can be time consuming and since the commonest approach is to sequence only the exons and flanking portions of the introns, the method could miss causal mutations in non-coding regions. In this study we used association-based exclusion analysis, a technique that requires only the use of animals affected with the condition and can be used for rapid exclusion of candidate loci. Obviously the hope is to identify a candidate locus that cannot be excluded and would thus warrant further investigation as the potential site of the disease causing mutation. Using association-based exclusion analysis we were able to exclude with high probability 11 candidate genes for OM in Cairn terriers.

We argue here that the assumptions (no locus or allelic heterogeneity, or recombination and/or new mutations, as listed in the Results section) that underlie this approach are appropriate. Giger and colleagues noted that, with very few exceptions, the causative mutations that have been identified for canine genetic diseases are breed specific or affect very closely related breeds [14]. The relative rarity of a Cairn terrier-ocular melanosis-like condition in any other breed of dog makes it very likely that this condition also resulted from a single mutation event.

We have estimated the probability of a recombination event causing a false exclusion of the true locus based upon the average recombination rate in dogs (1 cM/Mb). It has been suggested most chromosomal recombination occurs in “hotspots” in the human genome and 50% of all recombination occurs in less than 10% of the sequence [15]. In addition, recombination hotspots are relatively rare within or close to genes [16]. Although the region of linkage disequilibrium becomes smaller with increasing numbers of meiotic events (generations) after the founder mutation occurred, the timing of the first descriptions of ocular melanosis in Cairn terriers and the increasing incidence of glaucoma in the breed [17], suggests that the mutant allele became relatively common in the breed within recent time (probably less than 10 generations ago [2] and unpublished results). Thus, by using two markers, the average recombination rate becomes a conservative estimate, because of the small probability of the occurrence of two hotspots within or near a gene that would lead to a false exclusion.

Although it has been commented in several publications that some canine MS have observable mutations, to our knowledge no actual tabulation of mutation rate data has been published [18,19]. However, mutation rate data are available for humans, with the highest rates equivalent to about 1 mutation per 100 meioses [20]. We have used this highest human mutation rate as our estimation of the rate of mutation at our MS. Although it is possible that canine MS mutation rates could be greater than this estimate, rates in dogs as little as two-fold higher would likely have been previously detected and published. With the approach used here, two rare events (recombinations and/or marker mutations) would have to occur to falsely exclude the causative gene. We also note for exclusion analysis, recombinations and mutations at loci which do not contain the disease causing mutation do not alter the conclusions inferred from the analysis; the only concern is for violations of the underlying assumptions at the causal locus, leading to a false exclusion. With this approach and a single, necessary gene being responsible for the disease, most candidate genes can be screened in a relatively rapid and cost-effective manner. In this particular case, it is highly unlikely that the causal gene is among these 11 candidates.

## Conclusions

This study provides strong evidence to exclude 11 potential candidate genes from association with the Cairn terrier OM locus. Further studies are required to identify the gene mutation that is causal for this condition.

## Methods

### Animals

DNA was extracted by standard techniques from blood samples from privately owned pet Cairn terriers that had been diagnosed with OM by a veterinary ophthalmologist. The clinical information provided was reviewed by SPJ or JTB. All procedures were in compliance with the ARVO statement for the Use of Animals in Ophthalmic and Vision Research and covered by Michigan State University IACUC AUFs 05-08-076-00 or 05-11-106-00.

### Development of polymorphic markers for candidate genes

Candidate genes were selected because they either had a known involvement in ocular pigmentary disturbances in other species or a role in melanocytes and melanosome development.

Two polymorphic markers were developed for each gene (Table 1). Microsatellites (MS) were identified from the USCS Genome Browser ( http://genome.ucsc.edu/) May 2005 build of the canine genome using the repeat masker track. Single nucleotide polymorphism (SNP) markers were selected from the list of canine SNPs published by the Broad Institute of the Massachusetts Institute of Technology ( http://www.broadinstitute.org/mammals/dog). With the exception of *COMT* MS1 and *GPNMB* SNP2 all markers were within 600 kb from the furthest end of the candidate for which they were selected. Primers to amplify each marker by polymerase chain reaction were designed using the Primer3 program ( http://frodo.wi.mit.edu/primer3/). A universal labeling method was employed to fluorescently label the MS PCR products [21]. The MS markers were sized on an ABI PRISM 3130 genetic analyzer (Additional file 1: Table S1). Restriction enzyme digest assays were designed to allow genotyping of several of the SNPs (restriction enzymes shown in Table 1) and the other SNPs genotyped by direct sequencing of the PCR products (Additional file 1:Table S2).

### Calculation of probability of falsely excluding a genetic locus

Markers close to the causal gene mutation are likely to be identical by descent in all affected alleles unless a recombination event between marker and mutation occurred or the marker mutated. Either event could lead to falsely excluding the candidate locus. Therefore, the probability of a false exclusion of the causal gene due to such inconsistencies was calculated (see Additional file 2 for an example of calculations). We first counted the number of necessary recombination/mutation events to obtain the genotype that would result in a false exclusion of the true culprit gene. Then the greater of the two probabilities of a recombination and a marker mutation was used for subsequent calculations. In all cases, if a marker was a SNP or an insertion/deletion the probability of a recombination was used (i.e., a recurrent SNP and insertion/deletion mutation is assumed to be always rarer than the probability of a recombination event for the data reported here). The probability of a recombination event was assumed to be proportional to the distance of the marker to the end of the most distant exon. The recombination rate per unit of DNA is equivalent to 1 cM per Mb in the dog, based upon the estimates that the autosomal sex-averaged linkage map is 26.5 Morgans [22], and the physical map is 2631 Mb [23]. For all MS, the probability of a marker mutation was assumed to be 0.01, based upon the mutation rate for a canine MS set at the highest mutation rate seen among human MS [20]. The probabilities for each recombination or marker mutation event were then multiplied for both markers if they flanked the gene on opposite sides, or for the number of steps for a single marker if it was contained within the gene (note that for markers that do not flank both sides of a gene, it is possible for a single recombination to produce non-shared alleles for both markers, although the much tighter linkage for markers contained within a gene can often make up for this disadvantage). The probability was then adjusted for the estimated number of generations (independent chances for a recombination/mutation) since the foundation event; 1 – (1 – p)^n^, where n equals the estimated number of generations since the foundation event (in this case, ten generations – based on first descriptions of OM).

## Abbreviations

OM: Ocular melanosis;ASIP: Agouti signaling protein;COMT: Catechol-o-methyltransferase;GPNMB: Glycoprotein NMB;GSK3B: Glycogen synthase kinase 3-beta;LYST: Lysosomal trafficking regulator;MCIR: Melanocortin 1 receptor;MITF: Microphthalmia transcription factor;SILV: Silver;TYR: Tyrosinase;TYRP1: Tyrosinase related protein 1;TYRP2: Tyrosinase related protein 2

## Competing interests

The authors declare that they have no competing interests.

## Authors’ contributions

PAW: Investigated most of the candidate genes and helped write the paper. JTB: Reviewed clinical information for the OM-affected dogs and helped write the paper. CRQ: Investigated some of the candidate genes. PJV: Provided advice for exclusion analysis and calculated the probability of a false exclusion and helped write the paper. SMPJ: Obtained funding, selected most of the candidate genes, examined some affected dogs and reviewed clinical descriptions of affected dogs. Coordinated writing of the paper. All authors read and approved the final manuscript.

## Supplementary Material

Additional file 1: Table S1 Genotyping results for microsatellites on the ABI 3130 genetic analyzer. Dogs with one recombination or mutation event are boxed. Light gray boxes indicate a second recombination or mutation event. Dark gray boxes indicate a third recombination or mutation event. **Table S2.** Biallelic genotyping results. Biallelic genotyping results with SNP information, SNP # correlates with Table 1, SNP position. Click here for file

Additional file 2 **Supplementary Methods.** Example of p value calculations using microsatellite markers. Click here for file
