# Supplementary material for: Exclusion of eleven candidate genes for ocular melanosis in cairn terriers
Source: J Negat Results Biomed. 2013 Mar 1;12:6. doi: 10.1186/1477-5751-12-6 (PMC3599239; doi:10.1186/1477-5751-12-6)
Supplement: Additional file 2 — Supplementary Methods. Example of p value calculations using microsatellite markers. [file 1477-5751-12-6-S2.doc]

**Supplementary Methods:**

We provide here an example to show how the probability of a false exclusion is calculated. Note that we are calculating a statistic (what is the probability of an event causing a false exclusion?) rather that performing a statistical test for differences between two populations (e.g., what is the probability that allele frequencies are statistically different between affected and unaffected animals?). For LYST-1 (column 1, supplementary Table 1), ten affected dogs were genotyped for a microsatellite marker (which is a pentanucleotide - TTTTC repeat) that is 0.345 Mb upstream of the end of the farthest exon of the *LYST* candidate gene. Because we are excluding under a dominant model, all dogs would need to share at least one copy of the identical allele if this was the culprit gene. We assume that one bp differences in alleles are sizing errors in the ABI 3130 Genetic Analyzer and, therefore, that alleles that differ by one bp are identical. For example, the 449 bp alleles and 451 bp alleles found in dogs 1, 3 and 6, respectively, are considered to be identical to the 450 bp allele.

We also assume that an allele size difference of one repeat (5 bp for this pentanucleotide microsatellite) such as 445 bp allele (dog 5) and the 455 bp alleles (dogs 4 and 7) are caused by a single step mutation from the 450 bp allele, and thus must also be considered to be identical to the 450 bp allele. This is because an alteration of +/- one repeat may be a relatively common event based upon microsatellite mutation rates (Sun et al., 2012). All other alleles must be due to microsatellite mutations with two or more repeat units involved (occurring at a maximum frequency of 0.01) or recombination (which is proportional to the distance from the distal end of the gene divided by the probability of recombination (1% chance for each Mb).

For LYST-1 the first eight dogs (dogs 1 to 8) all share at least one 450 bp allele. However, two dogs, 9 and 10, do not share the most common allele of 450 bp. Therefore, the probability of falsely excluding this gene based upon this LYST-1 marker, if it was the true culprit, would be equal to the probability of either a microsatellite mutation or a recombination event causing the two dogs to have discordant genotypes. both dogs (9 and 10) share a 429 (or 430) allele, but only one mutation or recombination event is counted because this could be the same event that occurred in an ancestor that was inherited by both dogs (counting these as separate events would make the probability of a false exclusion even lower).

The distance of the LYST-1 marker is 0.345 Mb from the farthest exon of the *LYST* gene, and the probability of a recombination event is 0.345 Mb/(1 Mb/0.01 Morgan) = 3.45 x 10^-3^. The probability of a microsatellite mutation is assumed to be the highest possible (see the Discussion), which is 0.01. Therefore, we use the greater of these two values to calculate the probability of a false exclusion, which, in this case, is 0.01 for the first marker.

The second marker for the *LYST* candidate gene is LYST-2 (column 2, supplementary Table 1). The most common allele is 342 bp, shared by dogs 1, 2, 3, 9, and 10. The remaining dogs do not share this most common allele. The most common allele among these discordant dogs is 310 bp, and the 306 bp allele is considered to be identical to the 310 bp allele (as discussed above). Therefore, there must be two discordant alleles; 310 and either 303 or 315 (only one of these is counted as being a discordant allele because the calculations are done under a dominant model of inheritance).

The distance of this marker from the farthest exon of the gene is 0.368 Mb. The probability of a recombination event is 0.368 Mb/(1 Mb/0.01 Morgans) = 3.68 x 10^-3^. Because there must be two events causing discordances, these independent values are multiplied together: (3.68 x 10^-3^)^2^ = 1.35 x 10^-5^. The probability of two independent microsatellite mutations is (0.01)^2^ = 1 x 10^-4^. Because, in this case, the probability of a microsatellite mutation is greater than that for recombination, we use the more conservative value (i.e., 1 x 10^-4^ rather than 1.35 x 10^-5^).

Because the LYST-1 and LYST-2 bracket the *LYST* gene, the events that caused the discordances must be independent (e.g., any single recombination could not cause discordances for both markers with the causative mutation assumed to be in the coding region or nearby promoter region of the gene). Therefore, the total probability for falsely excluding *LYST* as the true culprit because of a meiotic marker mutation or recombination event occurring in a parent would be 0.01 x (1 x 10^-4^) = 1 x 10^-6^.

However, the mutation that lead to ocular melanosis is estimated to have occurred ten generations before the samples were collected, based upon the first known observation of OM in the breed, and assuming the average generation time of four years is the same for Cairn terriers as it is for the ten dog breeds studied by Neff and colleagues; (2004 – 1964 years)/4 years/generation = 10 generations (SPJ, personal observation; Neff et al., 2004). The increased probability for recombination or marker mutation over ten generations can be calculated in a manner similar to the reduction in linkage disequilibrium over this time (e.g., see Jorde, 1995). Thus, for a single recombination the probability is 1- (1 – (1 x 10^-6^))^10^ = 1 x 10^-5^.

Note also that because the mutation rate for a single nucleotide is about 1 x 10^-8^ per generation (assuming that canine mutation rates are similar to human mutation rates; Campbell et al., 2012), the probability for recombination causing a false exclusion will always be greater than a marker mutation (unless the marker is less than 100 bp from the mutation), and so the recombination rate, rather than the marker mutation rate, is always used for SNP markers (Campbell et al, 2012; and references contained therein).

**References:**

Campbell CD, Chong JX, Malig M, Ko A, Dumont BL, Han L, Vives L, O'Roak BJ,

Sudmant PH, Shendure J, Abney M, Ober C, Eichler EE. Estimating the human mutation rate using autozygosity in a founder population. Nat Genet. 2012 online Sep 23.

Jorde LB. Linkage disequilbrium and a gene-mapping tool. Am J Hum Genet 1995’56:11-14.

Neff MW, Robertson KR, Wong AK, Safra N, Broman KW, Slatkin M, Mealey KL,

Pedersen NC. Breed distribution and history of canine mdr1-1Delta, a pharmacogenetic mutation

that marks the emergence of breeds from the collie lineage. Proc Natl Acad Sci U S A. 2004,101:11725-30.

Sun JX, Helgason A, Masson G, Ebenesersdóttir SS, Li H, Mallick S, Gnerre S,

Patterson N, Kong A, Reich D, Stefansson K. A direct characterization of human mutation based on microsatellites. Nat Genet 2012, online Aug 23.
